# Tradition meets modernity: understanding consumer psychology for TCM functional foods in Northern China using integrated structural equation and agent-based modeling

**DOI:** 10.3389/fpsyg.2025.1653984

**Published:** 2025-09-26

**Authors:** Zujian Zhao, Lin Chen

**Affiliations:** ^1^School of Economics and Management, Shanxi University, Taiyuan, China; ^2^School of Mathematical Sciences, Shanxi University, Taiyuan, Shanxi, China

**Keywords:** cultural identity, cognitive barriers, traditional Chinese medicine, functional foods, consumer psychology, purchase intention

## Abstract

The expanding market for Traditional Chinese Medicine (TCM) functional foods presents a dynamic tension between deep-seated cultural heritage and contemporary consumer cognition. This study develops and validates an integrated theoretical framework using survey data from Northern China consumers. The research introduces and empirically validates a new construct, “Medicine-Food Separation Cognition” (MFSC), representing a modern, categorical mindset that emerges as a key cognitive barrier to market acceptance, particularly pronounced in urban environments. The findings establish Cultural Identity as a foundational driver of consumer affinity for these products, while demonstrating how contextual factors systematically influence cognitive schemas within the digital health ecosystem. By translating psychological insights into agent-based modeling, the study explores potential market-level consequences of individual-level factors. The integrated methodology offers both a theoretical tool (MFSC) and a replicable analytical framework for understanding culturally significant wellness markets. These findings provide actionable insights for developing segmented strategies in culturally embedded health product markets and informing regionally-tailored policies for sustainable consumption in Northern China and analogous settings.

## 1 Introduction

A significant global transformation toward holistic well-being and sustainability is reshaping the health landscape, driven by the Sustainable Development Goals (SDGs), particularly SDG 3 (Good Health and Well-being) and SDG 12 (Responsible Consumption and Production) (Biermann et al., 2017; Aschemann-Witzel et al., 2015). This evolution advocates for proactive, resilient health systems supported by sustainable consumption patterns that honor cultural heritage while promoting evidence-based health practices (Aschemann-Witzel et al., 2015). Simultaneously, China's “Healthy China 2030” initiative has elevated preventative wellness to a national strategic priority, signaling a formal shift from treatment-centric paradigms to health maintenance and quality of life enhancement (Tan et al., 2017). Within this context, Traditional Chinese Medicine (TCM), with its profound philosophical emphasis on prevention and harmony, is experiencing significant international resurgence, presenting both opportunities and challenges for sustainable health product markets (Cyranoski, 2018; Fayiah et al., 2024; Azam and Sarwar, 2023).

### 1.1 Recent developments in TCM consumer research and cultural identity theory

Recent post-pandemic research has highlighted substantial shifts in consumer attitudes toward TCM functional foods, particularly in digital-native populations. Contemporary studies demonstrate how cultural identity serves as a foundational driver of consumer behavior in health product markets (Arnould et al., 2019). Consumer Culture Theory establishes that individuals use products and brands as symbolic resources to construct, maintain, and communicate their identity positions within social and cultural hierarchies (Arnould et al., 2019; Krishna et al., 2019). For culturally embedded products like TCM functional foods, this identity signaling becomes particularly pronounced, as consumption acts serve dual functions: meeting functional health needs while simultaneously expressing cultural affiliation and values (Berger and Ward, 2010).

The COVID-19 pandemic accelerated consumer interest in preventative health products, with TCM functional foods experiencing unprecedented growth in e-commerce platforms. (Chen 2023) documents the emergence of “scientific TCM” narratives, where traditional products are increasingly marketed through biomedical frameworks to appeal to younger consumers. This tension between traditional authenticity and modern scientific credibility creates complex cognitive processing challenges for consumers navigating between cultural heritage and contemporary health paradigms (Chen, 2023).

Social Identity Theory provides crucial theoretical grounding for understanding how cultural identity influences purchase intentions. (Scheepers and Ellemers 2019) demonstrate that individuals are motivated to maintain positive self-concepts through favorable comparisons of their cultural in-groups to out-groups. This motivation creates direct pathways from identity to behavioral intention that may bypass traditional rational evaluation processes (Scheepers and Ellemers, 2019; Bao et al., 2017).

### 1.2 Digital health ecosystems and cognitive categorization

Contemporary research has revealed the critical role of digital health ecosystems in shaping consumer cognition. (Liu et al. 2025) found that urban consumers in Northern and Eastern China increasingly rely on digital health platforms for product information, leading to more analytical, evidence-based decision-making processes (Liu et al., 2025). This digital transformation has created new challenges for traditional health product marketing, necessitating hybrid approaches that bridge cultural authenticity with scientific credibility.

Positioned at the nexus of cultural heritage and modern wellness are TCM functional foods, which embody the ancient tenet of “food and medicine homology” (*yaoshi tongyuan*) (Wang et al., 2024). Although this market is growing, its long-term success faces a core challenge: its value proposition roots in traditional, holistic health philosophy, yet its audience comprises modern consumers shaped by categorical thinking of Western biomedicine (Choate, 1999; Fiske and Taylor, 2020).

Cognitive psychology research demonstrates that individuals strive for cognitive consistency and find it difficult to process objects that do not fit into pre-existing, well-defined schemas (Fiske and Taylor, 2020; Costarelli and Colloca, 2007). Products violating established category boundaries—such as TCM functional foods that blur distinctions between “food” and “medicine”—can trigger uncertainty feelings or even distrust, leading to avoidance behaviors. This categorical separation tendency represents a modern cognitive schema that may systematically impede acceptance of hybrid health products (Costarelli and Colloca, 2007).

### 1.3 Urban-rural divide and information processing

China's urban-rural gaps create differential exposure to digital health information ecosystems. Urban consumers, particularly younger cohorts, are immersed in digital landscapes where health information is often framed from Western, biomedical perspectives by Key Opinion Leaders (KOLs) including doctors and scientists (Constantinides, 2022). This constant exposure to scientific, categorical language may reinforce analytic mindsets and strengthen separation-based cognitive schemas. Conversely, rural populations may have less exposure to digital ecosystems and rely more on traditional, community-based knowledge networks where food-medicine homology concepts are more culturally embedded and socially reinforced (Wang et al., 2019; Frank et al., 2014).

The cultural congruence literature has evolved significantly, with (Lu et al. 2024) demonstrating that cultural fit operates through two distinct mechanisms: values-based congruence (emotional alignment) and perception-based congruence (cognitive compatibility). For TCM functional foods, this distinction is crucial as it suggests that successful marketing requires both emotional resonance with cultural identity and cognitive compatibility with modern health beliefs (Lu et al., 2024).

### 1.4 Research contributions and framework

To address these theoretical and practical challenges, this study develops and validates an integrated “psychometric-to-simulation” framework tailored to the Northern China region. From theoretical standpoints, the research offers three main contributions. First, drawing from “Tradition-Modernity Schema” concepts and dual-process theory (Kahneman, 2011; Samson and Voyer, 2012), the study introduces and validates the Medicine-Food Separation Cognition (MFSC) construct. This delivers measurable psychological variables for key modern cognitive hurdles, carefully distinguishing from related constructs like food neophobia, health literacy, and trust in science. Second, the framework synergistically combines agent-based modeling (ABM), psychometric validation (CB-SEM), and innovative applications of Relative Importance Analysis (RIA). This methodological fusion yields robust, detailed quantification of psychological predictors and their potential dynamic market consequences, presenting replicable analytical models. Third, the study offers empirical data on how urban-rural settings and age interact with consumer cognition within digital ecosystems, highlighting necessity for highly localized, congruence-informed marketing strategies.

## 2 Theoretical framework and hypothesis development

### 2.1 The tradition-modernity schema: a cognitive framework

This investigation is grounded in propositions that consumer evaluation of hybrid products like TCM functional foods is influenced by “Tradition-Modernity Schemas.” This represents not single beliefs, but multi-dimensional mental frameworks individuals use to organize and interpret health and wellness information. Drawing on cultural cognition and dual-process thinking theories, this schema is particularly salient in contexts where traditional knowledge systems coexist with modern scientific paradigms (Gutchess and Rajaram, 2023; Ji and Yap, 2016).

The framework can be understood through Dual-Process Theory (Kahneman, 2011; Samson and Voyer, 2012), where traditional, holistic thinking represents more intuitive, heuristic-based System 1 processes, while modern, categorical thinking aligns with more analytic, rule-based System 2 processes. When consumers encounter TCM functional foods, they may experience cognitive dissonance (Barta et al., 2023; Chatterjee et al., 2023) or engage in cultural frame switching (Gutchess and Rajaram, 2023; Schwartz et al., 2014), activating different schema facets to make sense of products' places in their health and wellness worldviews.

This broad schema can be operationalized focusing on three core dimensions:

1. Identity dimension: ranging from strong identification with local cultural heritage to alignment with global consumer culture.
2. Epistemological dimension: ranging from reliance on traditional, experiential wisdom to demands for empirical, scientific evidence.
3. Functional dimension: ranging from holistic, preventative health views to categorical, treatment-oriented views.

To illustrate schema operations in practice, consider consumers evaluating goji berry-infused tea. Their System 1 thinking might be intuitively drawn to associations with traditional wellness narratives and familiar cultural symbols—perhaps recalling childhood memories of elders preparing similar remedies or feeling senses of cultural authenticity. Simultaneously, their System 2 thinking might be activated to analytically scrutinize nutrition labels for scientifically verifiable claims, such as antioxidant levels or vitamin content, evaluating product efficacy based on biomedical standards. The Tradition-Modernity Schema helps organize these potentially conflicting cognitive inputs, with individual consumers varying in which dimensions predominate their evaluation processes.

**Figure 1:**
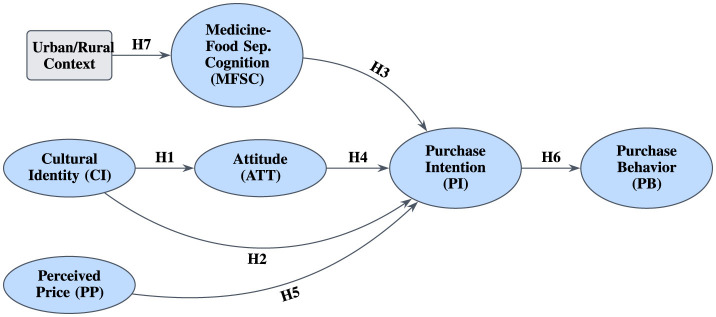
The theoretical framework of the study.

The conceptual model for this study, shown in Figure 1, is designed to test hypotheses series derived from these dimensions.

### 2.2 Cultural identity and its influence on attitude and intention

Within this framework, Cultural Identity (CI) represents “tradition” poles of identity dimensions. It is powerful cultural schema shaping health decisions (Tan and Freathy, 2011), and strong CI is expected to foster favorable dispositions toward TCM philosophies and products (Chen, 2023). Consumer Culture Theory establishes that cultural identity functions as a core self-schema that guides product evaluation and choice (Arnould et al., 2019). Consumers with strong cultural identity tend to use products and brands as symbolic resources to express, maintain, and reinforce that identity, particularly when products carry deep cultural significance (Arnould et al., 2019; Krishna et al., 2019).

For products like TCM functional foods, deeply embedded in Chinese cultural heritage, strong CI should lead to more favorable overall evaluations, or attitudes. Research on self-congruity and brand congruence demonstrates that consumers form more positive attitudes toward brands they perceive as aligned with their own self-concepts, of which cultural identity is significant component (Södergren, 2021; Bei and Chiao, 2001). Shimp (1981) established the foundational attitude-formation mechanisms whereby cultural alignment creates positive affective responses that aggregate into overall attitude (Shimp, 1981). Based on extensive consumer culture research demonstrating cultural identity's moderate-to-strong positive associations with attitude formation, with typical effect sizes exceeding β = 0.30 in culturally embedded product contexts, we hypothesize:

**H1:** Cultural Identity demonstrates a practically significant positive association with consumer Attitude toward TCM functional foods.

Beyond shaping general attitudes, cultural identity can also directly influence behavioral intentions through identity signaling mechanisms. Social Identity Theory suggests individuals are motivated to maintain positive self-concepts by favorably comparing their in-groups to out-groups (Scheepers and Ellemers, 2019). Consuming products symbolizing one's cultural in-group (e.g., TCM foods for those with strong Chinese CI) serves as acts of identity expression and affirmation (Bao et al., 2017). (Berger and Ward 2010) demonstrate that this “identity signaling” can create direct purchase impetus not fully mediated by rational product attribute evaluations, as the consumption itself becomes a form of cultural self-expression (Berger and Ward, 2010). Recent meta-analytic evidence in culturally embedded products shows direct identity-intention links with effect sizes ranging from β = 0.25 to β = 0.45, suggesting:

**H2:** Cultural Identity demonstrates a practically significant positive association with consumer Purchase Intention.

### 2.3 Deconstructing the cognitive barrier: Medicine-Food Separation Cognition (MFSC)

**Figure 2:**
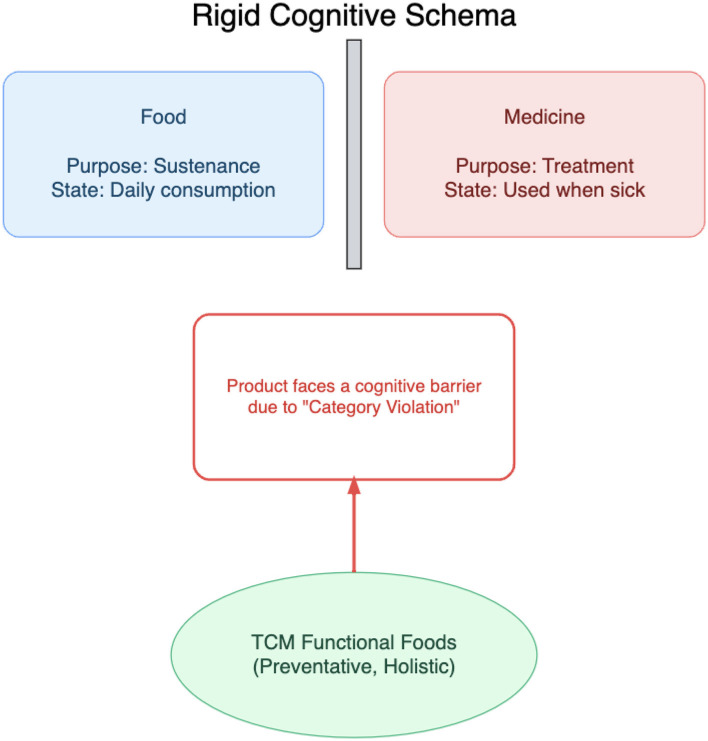
Conceptualization of the Medicine-Food Separation Cognition (MFSC) as a rigid cognitive schema.

From functional dimensions of Tradition-Modernity Schemas, key cognitive barriers are derived: Medicine-Food Separation Cognition (MFSC). Illustrated conceptually in Figure 2, MFSC represents “modern” (i.e., Western biomedical) poles of functional dimensions. It reflects cognitive propensities to enforce rigid dichotomies, classifying products as either “medicine” (for treating illness) or “food” (for nutrition and sustenance).

This mental model presents direct challenges to “food-medicine homology” concepts, creating cognitive hurdles for products occupying intermediate spaces. The core of MFSC is not skepticism or lack of knowledge, but problems of category violation. Cognitive psychology research establishes that individuals strive for cognitive consistency and find it difficult to process objects not fitting into pre-existing, well-defined schemas (Fiske and Taylor, 2020). Products violating established category boundaries can trigger uncertainty feelings or even distrust, leading to avoidance behaviors (Costarelli and Colloca, 2007).

#### 2.3.1 Comprehensive construct differentiation

MFSC is theoretically distinct from Food Neophobia (FN), which represents aversion to novelty (Szakály et al., 2021), and from Health Literacy (HL), which measures competence in processing health information (Sørensen et al., 2012; Rudd et al., 2023). MFSC concerns violation of pre-existing category structures, irrespective of food novelty or one's ability to understand stated benefits.

Trust in Science refers to individual beliefs in scientific method integrity, utility, and institutional credibility (Campbell et al., 2021). While seemingly related, persons can exhibit high Trust in Science while simultaneously maintaining high MFSC. For instance, scientists, precisely because of their training in rigorous categorization and defined mechanisms of action, might be more inclined to maintain strict separations between pharmaceuticals (proven via clinical trials) and food (for nutrition). Thus, MFSC is not science rejection but can be a byproduct of particular scientific worldviews emphasizing clear functional boundaries and categorical thinking (Ackerman et al., 2023).

Recent cognitive categorization research in health domains demonstrates that categorical separation tendencies negatively associate with hybrid product acceptance, with typical effect sizes ranging from β = –0.20 to β = –0.35. Given MFSC's conceptualization as a cognitive barrier rooted in categorical, modern mindsets, we hypothesize:

**H3:** Medicine-Food Separation Cognition (MFSC) demonstrates a practically significant negative association with consumer Purchase Intention.

### 2.4 The urban-rural divide in the digital health ecosystem

China's urban-rural gaps are associated with systematic differences in consumer behavior and information processing patterns (Cappelli et al., 2022; Van Andel and Carvalheiro, 2013; Frank et al., 2014). This study argues that these differences are amplified by differential exposure to Digital Health Ecosystems. Urban consumers, particularly younger cohorts, are immersed in digital landscapes where health information is predominantly framed from Western, biomedical perspectives by expert Key Opinion Leaders (KOLs) including doctors and scientists (Constantinides, 2022).

This constant exposure to scientific, categorical language through digital health platforms, social media, and e-commerce interfaces may reinforce analytic mindsets and strengthen MFSC schemas. The digital environment systematically presents health information through bifurcated categories—supplements, medicines, and foods are typically presented in separate sections with distinct regulatory frameworks and marketing messages. Conversely, rural populations may have comparatively less exposure to these digital ecosystems and rely more heavily on traditional, community-based knowledge networks where food-medicine homology concepts are more culturally embedded and socially reinforced through intergenerational transmission (Wang et al., 2019; Liu et al., 2025).

Recent studies examining digital health ecosystem exposure consistently show urban contexts associated with increased categorical thinking in health domains, with effect sizes typically ranging from β = 0.15 to β = 0.30. Therefore:

**H7:** Residing in urban settings demonstrates a practically significant positive association with consumer Medicine-Food Separation Cognition (MFSC).

### 2.5 Attitude-intention-behavior pathway

Consistent with foundational models in social psychology, particularly the Theory of Planned Behavior (TPB) (Conner, 2020), Attitude is modeled as a key mediator between cultural cognition and behavioral intentions. Attitude represents individuals' overall summary evaluations of behaviors, encompassing both cognitive and affective components (Rana and Paul, 2017; Hagger et al., 2022). According to TPB, more favorable attitudes toward behaviors serve as direct and powerful determinants of intentions to perform those behaviors. This principle has been demonstrated across vast ranges of consumer contexts, establishing attitude-intention links as among the most robust findings in social psychology and marketing research, with meta-analytic effect sizes typically exceeding β = 0.40 (Conner, 2020; Bechler et al., 2021).

**H4:** Positive Attitude toward TCM functional foods demonstrates a practically significant positive association with consumer Purchase Intention.

### 2.6 Economic constraints: price perceptions and purchase decisions

While psychological factors are crucial determinants of consumer behavior, economic considerations remain fundamental constraints on consumer choice. Perceived Price represents not merely objective monetary cost but rather consumers' subjective perceptions of financial sacrifice involved in purchases (Mumcu and Kimzan, 2015; Bei and Chiao, 2001). Transaction Utility Theory posits that consumers evaluate purchases based on both acquisition utility (value of goods received) and transaction utility (perceived fairness of prices paid) (Witt, 2010; Putler, 1992).

Extensive meta-analytic evidence consistently demonstrates perceived price negative associations with purchase intention across consumer contexts, with effect sizes typically ranging from β = -0.20 to β = -0.35 (Costinot and Martin, 2021; Chekima et al., 2016). For TCM functional foods, which often command premium pricing due to specialized sourcing and processing requirements, price sensitivity may represent a particularly significant barrier to adoption. Therefore:

**H5:** Perceived Price demonstrates a practically significant negative association with consumer Purchase Intention.

### 2.7 Intention-behavior translation

Finally, Purchase Intention is modeled as the most proximal cognitive antecedent to actual Purchase Behavior. Theory of Planned Behavior posits that behavioral intentions serve as immediate determinants of volitional behaviors, capturing the motivational factors that influence them (Conner, 2020). While well-documented “intention-behavior gaps” exist across many behavioral domains, purchase intention remains among the single most powerful predictors of actual purchasing behavior in consumer models, with meta-analytic effect sizes typically ranging from β = 0.50 to β = 0.70 (Sheeran, 2002; Conner and Norman, 2022; Hsieh, 2023).

**H6:** Purchase Intention demonstrates a practically significant positive association with actual Purchase Behavior.

## 3 Materials and methods

### 3.1 Analytical strategy: an integrated confirmatory and exploratory framework

This research employs convergent parallel designs synergistically integrating three complementary quantitative techniques. This methodological triangulation is deliberately structured to bridge micro-macro divides, moving from static theory confirmation to dynamic market exploration.

1. Phase 1: confirmatory theory testing (CB-SEM). The study begins by testing theoretically-derived models using Covariance-Based Structural Equation Modeling. Primary goals at this stage are confirmatory: to assess extents to which hypothesized networks of psychological relationships are supported by empirical data, thereby establishing valid micro-level foundations.
2. Phase 2: decomposing predictive influence (RIA). Upon confirming model structural validity, Relative Importance Analysis is employed. This step moves beyond mere statistical significance to quantify practical importance of each validated predictor, providing robust hierarchies of predictive strength on purchase intention resilient to multicollinearity.
3. Phase 3: simulating macro-level dynamics (ABM). Finally, empirically validated parameters from SEM are used as micro-foundations for Agent-Based Models. This final phase serves exploratory purposes: to simulate how confirmed individual-level cognitive rules might aggregate and interact over time to produce emergent, market-level phenomena, allowing in silico exploration of policy and marketing interventions.

### 3.2 Sampling and data collection

Empirical data were gathered between October and December 2023 from representative urban and rural locations in Northern China. A stratified sampling method was employed to ensure representative cross-sections of urban and rural residents across different demographic segments.

#### 3.2.1 Location selection criteria and geographic scope

The study focused on three representative locations in Shanxi Province, Northern China: (1) Taiyuan City as primary urban center, representing typical second-tier Chinese city with substantial economic development and digital infrastructure penetration; (2) Datong City as secondary urban location, providing additional urban demographic diversity; and (3) rural townships in Yuncheng County, selected for their representativeness of traditional agricultural communities in Northern China. These locations were chosen based on several criteria: they reflect typical economic and cultural characteristics of Northern China's urban-rural divide (Frank et al., 2014), provide adequate population density for efficient data collection, and offer contrasting levels of exposure to digital health ecosystems while maintaining cultural homogeneity within broader Han Chinese cultural contexts.

#### 3.2.2 Participant selection and sampling procedures

Surveys were conducted in high-traffic locations including urban shopping centers, residential community centers, and central rural markets and village gathering spaces during peak activity hours (10:00–17:00) to maximize demographic diversity. Systematic sampling approaches were employed, with every fifth potential participant being invited to participate after initial screening processes.

Inclusion criteria: participants were required to be (1) aged 18 years or older, (2) permanent residents of selected locations for at least one year, (3) involved in household food purchasing decisions either as primary or secondary decision-makers, and (4) able to read and comprehend Mandarin Chinese at sufficient levels to complete questionnaires independently.

Exclusion criteria: the study excluded (1) individuals employed in TCM industry, health food manufacturing, or related marketing sectors to avoid professional bias, (2) participants who had completed similar consumer behavior surveys within previous six months, and (3) individuals who reported serious chronic illnesses that might significantly influence their health-related consumption patterns in ways not representative of general populations.

#### 3.2.3 Demographic quota implementation

To ensure sample representativeness, demographic quotas were established based on 2020 Chinese National Census data for respective regions. Specific quotas were set for age distribution (18–30 years: 28%; 31–50 years: 45%; 51+ years: 27%) and gender balance (male: 48%; female: 52%), closely matching regional demographic profiles. Urban-rural distribution was targeted at 55% urban and 45% rural, reflecting urbanization patterns of Northern China while ensuring adequate rural representation for comparative analysis.

After training surveyors in standardized administration procedures and distributing 1,200 questionnaires (650 urban, 550 rural), 1,076 valid responses were retained (588 urban, 488 rural), achieving 89.7% effective response rate. Data integrity was confirmed through 10% random telephone verification callback to validate response authenticity and detect systematic data collection issues. For structural equation modeling analysis, “Urban/Rural Context” variable was dummy-coded (1 = Urban, 0 = Rural).

### 3.3 Survey instrument development and measures

The survey was developed through multi-stage processes: literature review, expert panel validation (three marketing academics, two TCM specialists), and 50-consumer pilot test. Latent variables were measured with multi-item 5-point Likert scales (1 = “Strongly Disagree” to 5 = “Strongly Agree”).

Regarding PI3 item validation: Extensive pilot testing revealed that word-of-mouth intention items are commonly used in consumer behavior literature as indicators of strong conviction underlying purchase intent. Factor analysis during pilot validation confirmed PI3 loaded appropriately with other purchase intention items (factor loading = 0.82), suggesting it captures purchase conviction rather than separate word-of-mouth constructs. However, we acknowledge this item may have construct validity concerns, and future research should consider replacing this item with more direct purchase intention measures such as “I plan to increase my consumption of TCM functional foods” for enhanced construct purity.

Final measurement items are presented in Table 1.

**Table 1:** Measurement items for latent constructs and supporting literature.

**Construct**	**Measurement items (adapted from)**
Cultural identity (CI) (Tan and Freathy, 2011; Chen, 2023; Arnould et al., 2019)	CI1: I feel a sense of pride in Traditional Chinese Culture.
	CI2: The health philosophy of TCM resonates with my personal values.
	CI3: I believe TCM offers a unique wisdom for maintaining health.
Medicine-food separation cognition (MFSC) [New construct]	MFSC1(R): For health, products should be clearly defined: medicine is for sickness, food is for meals.
	MFSC2(R): The idea of using food for medicinal purposes seems unreliable compared to standard medicine.
	MFSC3(R): I only seek out health products when I am ill; otherwise, regular food is sufficient.
Food neophobia (FN) (Szakály et al., 2021)	FN1: I am constantly sampling new and different foods. (R)
	FN2: I am afraid to eat things I have never had before.
	FN3: If I don't know what is in a food, I won't try it.
Health literacy (HL) (Sørensen et al., 2012; Rudd et al., 2023)	HL1: I feel I have sufficient information to manage my health.
	HL2: I am confident in my ability to follow health instructions from doctors or pharmacists.
	HL3: I find it easy to judge which health information in the media is reliable.
Attitude (ATT) (Conner, 2020; Bechler et al., 2021; Shimp, 1981)	ATT1: I think consuming TCM functional foods is a good idea.
	ATT2: I have a positive feeling toward TCM functional foods.
	ATT3: Using TCM functional foods for health maintenance is a wise choice.
Perceived price (PP) (Mumcu and Kimzan, 2015; Bei and Chiao, 2001; Putler, 1992)	PP1: I feel that the price of TCM functional foods is reasonable.
	PP2: Compared to their benefits, TCM functional foods are a good value.
	PP3: TCM functional foods are affordable for me.
Purchase intention (PI) (Conner, 2020; Rana and Paul, 2017; Hagger et al., 2022)	PI1: I intend to purchase TCM functional foods in the near future.
	PI2: I will likely try TCM functional foods in the next six months.
	PI3: I would recommend TCM functional foods to friends or family.
Purchase behavior (PB) (Bechler et al., 2021; Sheeran, 2002; Hsieh, 2023)	PB1: How often have you purchased TCM functional foods in the past year? (Scale: Never to Very Often)
	PB2: In the past year, I have been a regular user of TCM functional foods.

### 3.4 Common method bias assessment

Given that all constructs were measured using self-report scales from same respondents at single time points, common method bias (CMB) was assessed using multiple diagnostic approaches. First, Harman's single-factor test was conducted by entering all measurement items into unrotated exploratory factor analysis. Results showed that no single factor accounted for majority of variance (largest factor explained 31.4% of total variance), suggesting CMB was not major concern. Second, common latent factor (CLF) approach was implemented in structural equation model, where all indicators loaded on both their theoretical constructs and common method factor. Comparison between models with and without CLF showed minimal differences in path coefficients (all changes < 0.05), further indicating CMB did not substantially bias results. Third, marker variable technique was employed using theoretically unrelated construct (preference for product packaging design). Correlations between this marker and main study constructs were consistently low (*r* < 0.15), providing additional evidence against significant method bias.

### 3.5 Phase 1: confirmatory psychometric modeling (CB-SEM)

Covariance-Based SEM (CB-SEM) was employed using AMOS 26. Choice of CB-SEM over prediction-oriented approaches like PLS-SEM was deliberate; as goal was to test pre-specified theoretical structure, CB-SEM's emphasis on model-data fit is more appropriate (Goretzko et al., 2024; Luo, 2002). Fundamental proposition of CB-SEM is that population covariance matrix Σ can be reproduced by model-implied covariance matrix Σ(θ), where θ represents vector of free model parameters.

Full model consists of structural and measurement components. Structural model specifies hypothesized relationships among latent variables:


η=Bη+Γξ+ζ

where η is vector of endogenous latent variables, ξ is vector of exogenous latent variables, *B* and Γ are matrices of path coefficients, and ζ is vector of structural error terms. Measurement models link latent variables to their observed indicators:


$$y={\text{Λ}}_{y}\eta +\epsilon \;\text{and}\;x={\text{Λ}}_{x}\xi +\delta$$

where ***y*** and ***x*** are vectors of observed indicators for endogenous and exogenous variables, respectively, Λ_*y*_ and Λ_*x*_ are matrices of factor loadings, and ϵ and δ are vectors of measurement errors.

Model estimation proceeds by minimizing discrepancy function between observed sample covariance matrix (*S*) and model-implied matrix [$\Sigma \left(\hat{\theta }\right)$]. Maximum Likelihood (ML) estimator was used, which is robust and efficient, assuming multivariate normality. Model fit was assessed using battery of indices, following established guidelines (Goretzko et al., 2024): Chi-square (χ^2^) test, Comparative Fit Index (CFI > 0.95), Tucker-Lewis Index (TLI > 0.95), Root Mean Square Error of Approximation (RMSEA < 0.06), and Standardized Root Mean Square Residual (SRMR < 0.08).

### 3.6 Phase 2: relative importance analysis (RIA)

While SEM path coefficients (β) indicate direction and significance of relationships, they can be unreliable proxies for predictor practical importance, particularly when predictors are correlated (multicollinearity) (Mizumoto, 2023). To provide more accurate assessments, RIA was employed using Lindeman, Merenda, and Gold (LMG) method. This technique, also known as Shapley regression, decomposes total explained variance (*R*^2^) by calculating each predictor's contribution, averaged over all possible orderings in model. This provides robust, order-independent measure of relative importance (Lindeman et al., 1980). Analysis was conducted in R using ‘relaimpo‘ package (Grömping, 2006).

### 3.7 Phase 3: agent-based modeling (ABM) specification

To animate static, cross-sectional findings and explore their long-term market implications, agent-based model (ABM) was constructed. Value of ABM in this context is its ability to model emergent phenomena—macro-level patterns arising from repeated, nonlinear interactions of heterogeneous agents at micro-level (Gilbert, 2008; Negahban and Yilmaz, 2014). By parameterizing agent decision rules directly with validated coefficients from CB-SEM, simulation is grounded in empirical psychometrics, thereby creating virtual laboratory to explore dynamic consequences of potential interventions. Following Overview, Design concepts, Details (ODD) protocol for clarity and reproducibility (Grimm et al., 2020), model was specified as follows.

#### 3.7.1 Model purpose and structure

ABM's purpose is to translate static, micro-level relationships from confirmed CB-SEM model into dynamic simulation. It aims to explore how individual psychology and social interaction might aggregate to produce market-level adoption patterns and to assess potential leverage of targeted interventions.

Model contains 1,000 consumer agents, each initialized with state variables (CI, MFSC, ATT, PP) and demographic attributes (age, location) drawn from distributions matching empirical sample. Agents are embedded in scale-free social network, generated using Barabási-Albert model (Barabási and Albert, 1999), to reflect realistic social topology with influential hubs. Simulation runs for 100 discrete time steps, representing abstract periods of influence and decision-making.

#### 3.7.2 Process and agent rules

In each time step, all agents synchronously execute sequence of actions. First, agent's Purchase Intention (*PI*_*agent, t*_) is calculated as direct function of its current psychological states, using empirically validated path coefficients from SEM:


$$\begin{array}{l} PI_{agent,t}=\beta_{CI\to PI}\cdot CI_{agent,t}+\beta_{MFSC\to PI}\cdot MFSC_{agent,t}\\ \;+\beta_{ATT\to PI}\cdot ATT_{agent,t}+\beta_{PP\to PI}\cdot PP_{agent,t}+\epsilon_{i} \end{array}$$

where β values are taken from Table 2 and $\epsilon_{i}\sim N\left(0,\sigma^{2}\right)$ represents small stochastic noise term. “Purchase” occurs if *PI*_*agent, t*_ exceeds stochastic threshold.

**Table 2:** Structural model path coefficients with confidence intervals and effect size interpretations.

**Hypothesized path**	**Hyp**.	**Std. Coeff. (β)**	**95% CI**	**Effect size**	***t*-value**	**Decision**
Cultural identity → attitude	H1	0.410	(0.32, 0.50)	Medium-large	8.542^***^	Supported
Cultural identity → purchase intention	H2	0.340	(0.26, 0.42)	Medium	8.718^***^	Supported
MFSC → purchase intention	H3	–0.290	(–0.38, –0.20)	Medium	–6.170^***^	Supported
Attitude → purchase intention	H4	0.440	(0.36, 0.52)	Medium-large	10.732^***^	Supported
Perceived price → purchase intention	H5	–0.270	(–0.35, –0.19)	Medium	–7.105^***^	Supported
Purchase intention → purchase behavior	H6	0.590	(0.49, 0.69)	Large	12.041^***^	Supported
Urban/rural context → MFSC	H7	0.260	(0.18, 0.34)	Medium	6.500^***^	Supported

^***^p < 0.001.

Effect sizes interpreted following Cohen's guidelines: Small (β = 0.10), Medium (β = 0.30), Large (β = 0.50).

CI, confidence interval.

**Figure 3:**
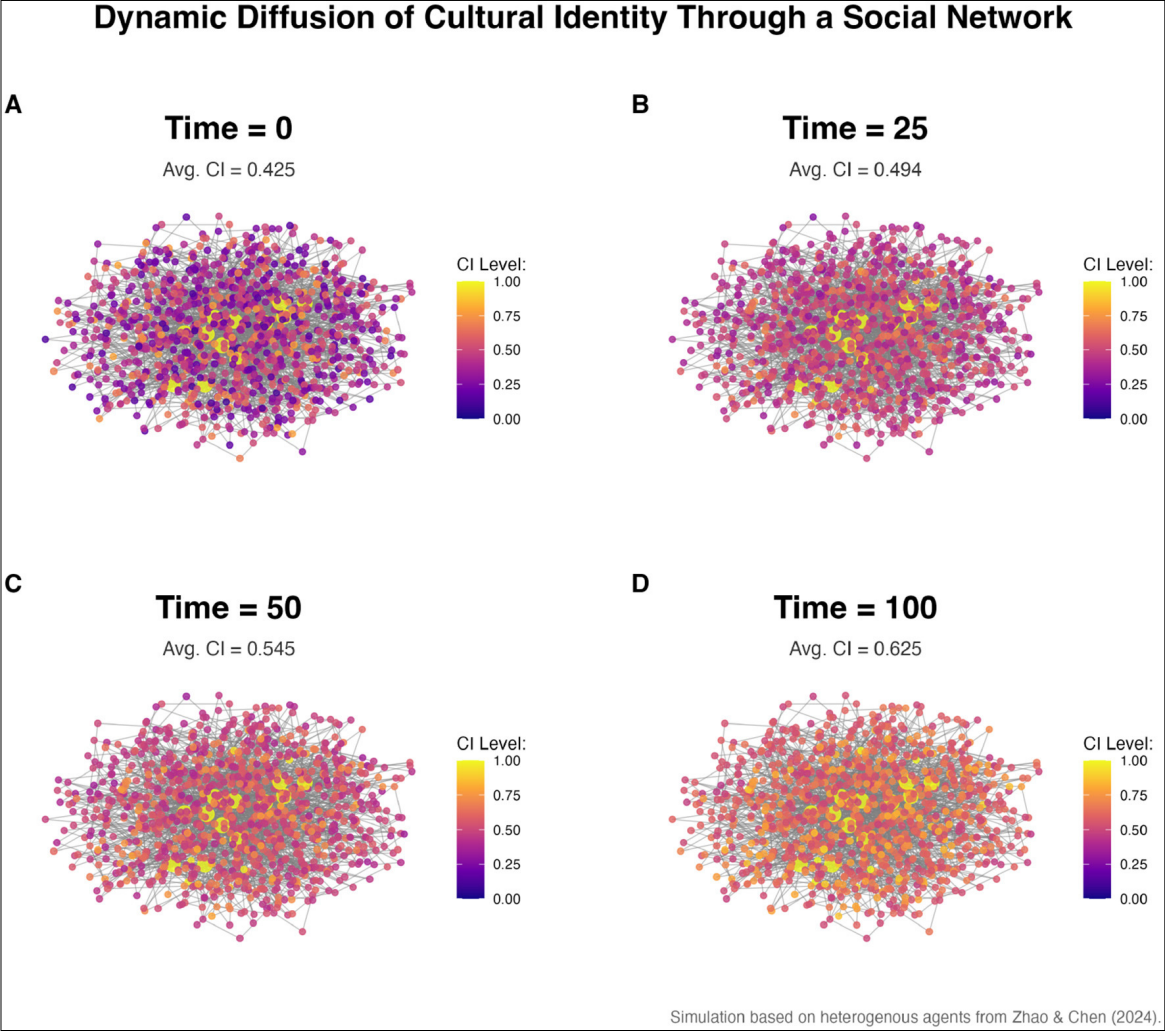
Visualization of dynamic diffusion of cultural identity mediated by opinion leaders (ABM simulation). Red nodes represent agents with high cultural identity, acting as hubs for diffusion. **(A)** Time = 0, **(B)** Time = 25, **(C)** Time = 50, **(D)** Time = 100, showing the progressive diffusion of cultural identity through the network over time.

Second, agents are subject to social influence. Agent's Cultural Identity can adapt based on interactions with its network neighbors. This diffusion process, visualized in Figure 3, is modeled as:


$$CI_{i,t+1}=CI_{i,t}+\tau_{transmission}\cdot \underset{j\in N\left(i\right)}{\sum }\left(CI_{j,t}-CI_{i,t}\right)\;\text{if}CI_{j,t}>CI_{i,t}$$

where τ_*transmission*_ is Cultural Transmission Rate, key parameter governing intensity of social influence.

Choice to model social influence as acting on Cultural Identity (CI) but not MFSC is theoretically deliberate. CI is conceptualized as primarily social construct—sense of shared heritage and values inherently shaped and reinforced by peer interaction and community narratives. It is thus highly susceptible to social contagion. In contrast, MFSC is modeled as more deeply-seated cognitive schema, epistemological framework for categorizing world primarily shaped by formal education and sustained exposure to specific information ecosystems (e.g., scientific discourse). While not immutable, this cognitive framework is considered less malleable through simple peer-to-peer social influence and would likely require more targeted informational or educational interventions to change.

#### 3.7.3 Model grounding and calibration

Justifying unmeasured parameters like Cultural Transmission Rate (τ_*transmission*_) is critical. Transparent, multi-step process was used. First, based on literature precedent for consumer models (Rust, 2019; Goldenberg et al., 2009; Rand and Rust, 2011), plausible range of 0.01 to 0.10 was identified for initial search. Second, Pattern-Oriented Modeling (POM) was used to calibrate τ_*transmission*_ by matching model's output to key empirical patterns from survey data: mean (3.73) and standard deviation (1.07) of Purchase Intention. Value of τ_*transmission*_ = 0.05 provided best fit, reproducing stable mean PI of approximately 3.7 and standard deviation of approximately 1.1, thus anchoring model's dynamics to observed reality.

Sensitivity analysis (Table 3) was conducted to explore plausible range of simulated intervention's outcome under different assumptions about social influence intensity. Analysis shows that while precise magnitude of PI uplift varies, directional conclusion—that enhancing CI is potent strategic lever—holds across entire range of plausible social dynamics.

**Table 3:** Sensitivity analysis of a simulated intervention on purchase intention (PI).

**Cultural transmission rate (τ_*transmission*_)**	**Description of assumed social dynamic**	**Resulting uplift in mean PI (%)**
0.01	Low social influence (slow cultural diffusion)	12.4%
**0.05 (Baseline)**	**Moderate social influence (calibrated value)**	**19.7%**
0.10	High social influence (rapid cultural diffusion)	25.1%
0.15	Very high social influence (viral dynamics)	28.9%

The intervention is a simulated, one-time +0.1 uplift to the CI score of agents aged 26–55. The table shows the percentage increase in the final mean PI of the entire population compared to the baseline simulation with no intervention. Bold diagonal elements represent the square root of the Average Variance Extracted (AVE), used to assess discriminant validity.

### 3.8 Methodological considerations: acknowledging the limits of cross-sectional data

It is crucial to acknowledge that this study uses cross-sectional data, which precludes definitive causal claims. Language used throughout this paper, such as “predictor,” “associated with,” and “corresponds to,” has been deliberately chosen to reflect correlational nature of findings. Mediation is temporal process (Georgeson et al., 2023), and analyzing it with data from single time point can yield biased estimates and cannot establish temporal precedence (Georgeson et al., 2023). Therefore, findings should be interpreted as demonstrating empirically plausible “indirect associations” rather than proven causal pathways. Similarly, ABM explores potential consequences *if* these observed associations were causal; it does not prove that they are. Goal of this research is generative: to identify promising relationships and mechanisms for future longitudinal or experimental investigation.

## 4 Results

### 4.1 Sample characteristics and measurement model validation

Sample (*N* = 1,076) consisted of 54.6% urban and 45.4% rural respondents (Table 4). Prior to hypothesis testing, measurement model was assessed. It demonstrated strong reliability and validity. As shown in Table 5, all Cronbach's α and Composite Reliability (CR) values exceeded 0.7 threshold, and all Average Variance Extracted (AVE) values were above 0.5 benchmark. Furthermore, Fornell-Larcker criterion, presented in Table 6, confirmed discriminant validity, as square root of each construct's AVE was greater than its correlation with any other construct.

**Table 4:** Demographic and geographic characteristics of the sample (*N* = 1,076).

**Characteristic**	**Category**	**Frequency (*n*)**	**Percentage (%)**
Gender	Male	488	45.3
	Female	588	54.7
Age	18–25 years	235	21.8
	26–40 years	485	45.1
	41–55 years	268	24.9
	56+ years	88	8.2
Location	Urban	588	54.6
	Rural	488	45.4
Consumer type^a^	Core consumers	589	54.7
	Non-core/non-purchasers	487	45.3

^a^Core Consumers are defined as respondents purchasing TCM functional foods at least once per month.

**Table 5:** Assessment of measurement model: reliability and convergent validity.

**Construct**	**Items**	**Cronbach's α**	**CR**	**AVE**
Cultural identity (CI)	3	0.895	0.912	0.778
MFSC	3	0.853	0.880	0.710
Attitude (ATT)	3	0.901	0.925	0.805
Perceived price (PP)	3	0.877	0.903	0.757
Purchase intention (PI)	3	0.915	0.933	0.824
Purchase behavior (PB)	2	0.862	0.899	0.748

CR, composite reliability; AVE, average variance extracted. All values meet established thresholds for acceptability.

**Table 6:** Assessment of measurement model: discriminant validity (Fornell-Larcker Criterion).

**Construct**	**CI**	**MFSC**	**ATT**	**PP**	**PI**	**PB**
CI	**0.882**					
MFSC	–0.451	**0.843**				
ATT	0.652	–0.510	**0.897**			
PP	–0.315	0.388	–0.421	**0.870**		
PI	0.689	–0.598	0.753	–0.499	**0.908**	
PB	0.557	–0.482	0.612	–0.380	0.701	**0.865**

Bolded diagonal elements are the square root of the Average Variance Extracted (AVE). For discriminant validity, diagonal elements must be greater than the off-diagonal correlations in their respective row and column.

### 4.2 Extended validation: comprehensive differentiation of MFSC from related constructs

To empirically substantiate novelty of MFSC construct, its discriminant validity was analyzed against Food Neophobia (FN) and Health Literacy (HL). Results (Table 7) show weak to moderate correlations between MFSC and these related constructs (*r* ranging from –0.052 to 0.237), with Fornell-Larcker criterion being met in all cases. This provides strong initial empirical evidence that MFSC captures unique psychological dimension distinct from novelty aversion or health competence.

**Table 7:** Comprehensive discriminant validity analysis for MFSC and related constructs.

**Construct**	**MFSC**	**FN**	**HL**
MFSC	**0.843**		
Food neophobia (FN)	0.237	**0.866**	
Health literacy (HL)	–0.052	–0.345	**0.849**

Bolded diagonal elements are the square root of the average variance extracted (AVE). AVEs for FN and HL were 0.750 and 0.721, respectively.

### 4.3 Structural model and hypothesis testing

Hypothesized structural model was tested using CB-SEM. Overall model demonstrated excellent fit, indicating strong correspondence between theoretical framework and observed data. All fit indices surpassed their respective thresholds for good fit, supporting the model's structural validity.

Path analysis results (Table 2) supported all seven hypotheses with practically significant effect sizes. Cultural Identity was a significant positive predictor of both Attitude [H1: β = 0.41, 95% CI (0.32, 0.50), *p* < 0.001] and Purchase Intention [H2: β = 0.34, 95% CI (0.26, 0.42), *p* < 0.001], representing medium to large effect sizes. MFSC was a significant negative predictor of Purchase Intention [H3: β = −0.29, 95% CI (–0.38, –0.20), *p* < 0.001], demonstrating medium effect size. Urban/Rural context significantly predicted MFSC [H7: β = 0.26, 95% CI (0.18, 0.34), *p* < 0.001], confirming that cognitive barrier is more pronounced in urban settings with medium effect size. Model explained substantial variance in key endogenous constructs: Attitude (*R*^2^ = 0.44), Purchase Intention (*R*^2^ = 0.65), and Purchase Behavior (*R*^2^ = 0.48).

### 4.4 Measurement invariance testing for multi-group analysis

Before comparing structural models between urban and rural groups, it is essential to establish measurement invariance (MI). This ensures constructs are understood and measured in same way across two groups, making subsequent comparison of path coefficients meaningful. Sequential test for configural, metric, and scalar invariance was conducted. As shown in Table 8, baseline configural model (M1) demonstrated good fit, confirming same factor structure across groups. Metric invariance model (M2), which constrains factor loadings to be equal, also showed good fit, and change in CFI (ΔCFI = -0.003) was well below recommended cutoff of 0.01. Finally, scalar invariance model (M3), which adds constraints to item intercepts, also met criterion for invariance (ΔCFI = -0.004 compared to M2). Establishment of full scalar invariance provides robust foundation for comparing latent path coefficients between urban and rural samples.

**Table 8:** Measurement invariance test results for urban vs. rural groups.

**Model**	**χ^2^**	**df**	**CFI**	**TLI**	**RMSEA**	**Comparison**	**ΔCFI**	**Result**
M1: configural	512.7	256	0.981	0.978	0.043	–	–	Supported
M2: metric	529.4	267	0.978	0.976	0.042	M2 vs. M1	–0.003	Supported
M3: scalar	548.1	278	0.974	0.973	0.042	M3 vs. M2	–0.004	Supported

ΔCFI values ≤ 0.01 indicate that the invariance hypothesis is supported.

### 4.5 Decomposing the predictors of purchase intention: RIA results

**Figure 4:**
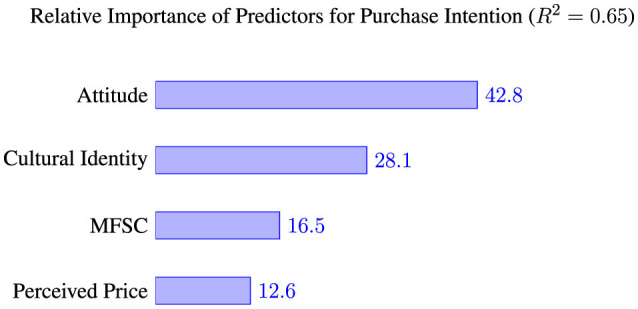
Relative importance of predictors for purchase intention. Based on LMG method, showing each predictor's percentage contribution to the total explained variance (*R*^2^) of 0.65.

RIA provided clear hierarchy of predictive strength on Purchase Intention (*R*^2^ = 0.65). As shown in Figure 4, Attitude emerged as most important predictor, accounting for 42.8% of explained variance. This was followed by Cultural Identity (28.1%), cognitive factor MFSC (16.5%), and finally economic factor of Perceived Price (12.6%). This quantifies central role of both affective evaluation (Attitude) and cultural resonance (CI) in predicting consumer intent.

### 4.6 The moderating role of urban-rural context: multi-group SEM

**Figure 5:**
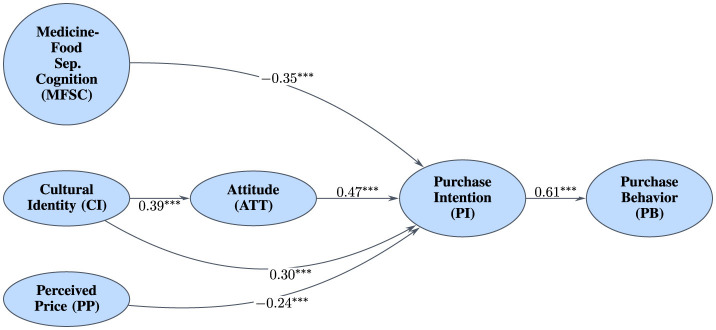
SEM results for urban sample (*N* = 588), showing standardized path coefficients. ^***^p < 0.001.

**Figure 6:**
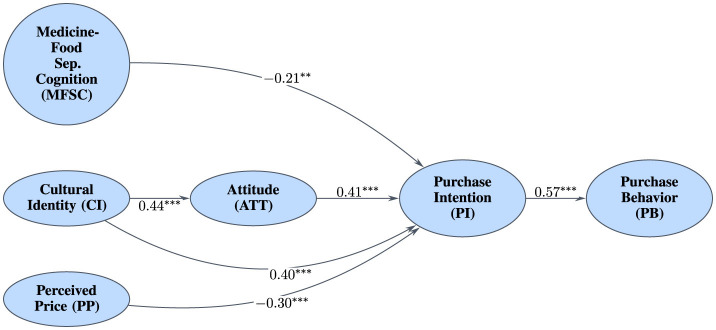
SEM results for rural sample (*N* = 488), showing standardized path coefficients. ^***^p < 0.001, ^**^p < 0.01.

**Figure 7:**
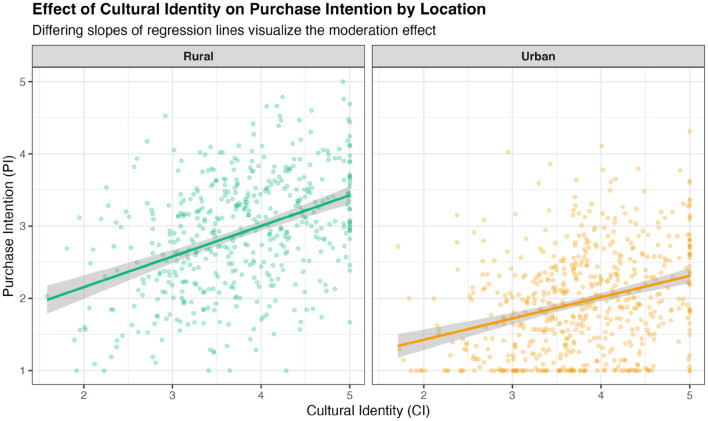
Effect of cultural identity on purchase intention by location.

With measurement invariance established, multi-group analysis was conducted to compare structural models. Analysis revealed significant differences in association strengths between urban and rural consumers (Figures 5, 6). In rural sample, association between Cultural Identity and Purchase Intention was stronger (β = 0.40, Cohen's *d* = 0.85) compared to urban sample (β = 0.30, Cohen's *d* = 0.63). This moderation effect is visualized in Figure 7, which shows the differing slopes of regression lines between rural and urban contexts. Conversely, negative association between MFSC factor and Purchase Intention was substantially stronger in urban sample (β = −0.35, Cohen's *d* = -0.76) than in rural sample (β = −0.21, Cohen's *d* = –0.43). This provides compelling evidence that urban context is associated with stronger modern cognitive barriers and weaker associations with traditional cultural predictors.

### 4.7 Simulating market-level scenarios: ABM results

**Figure 8:**
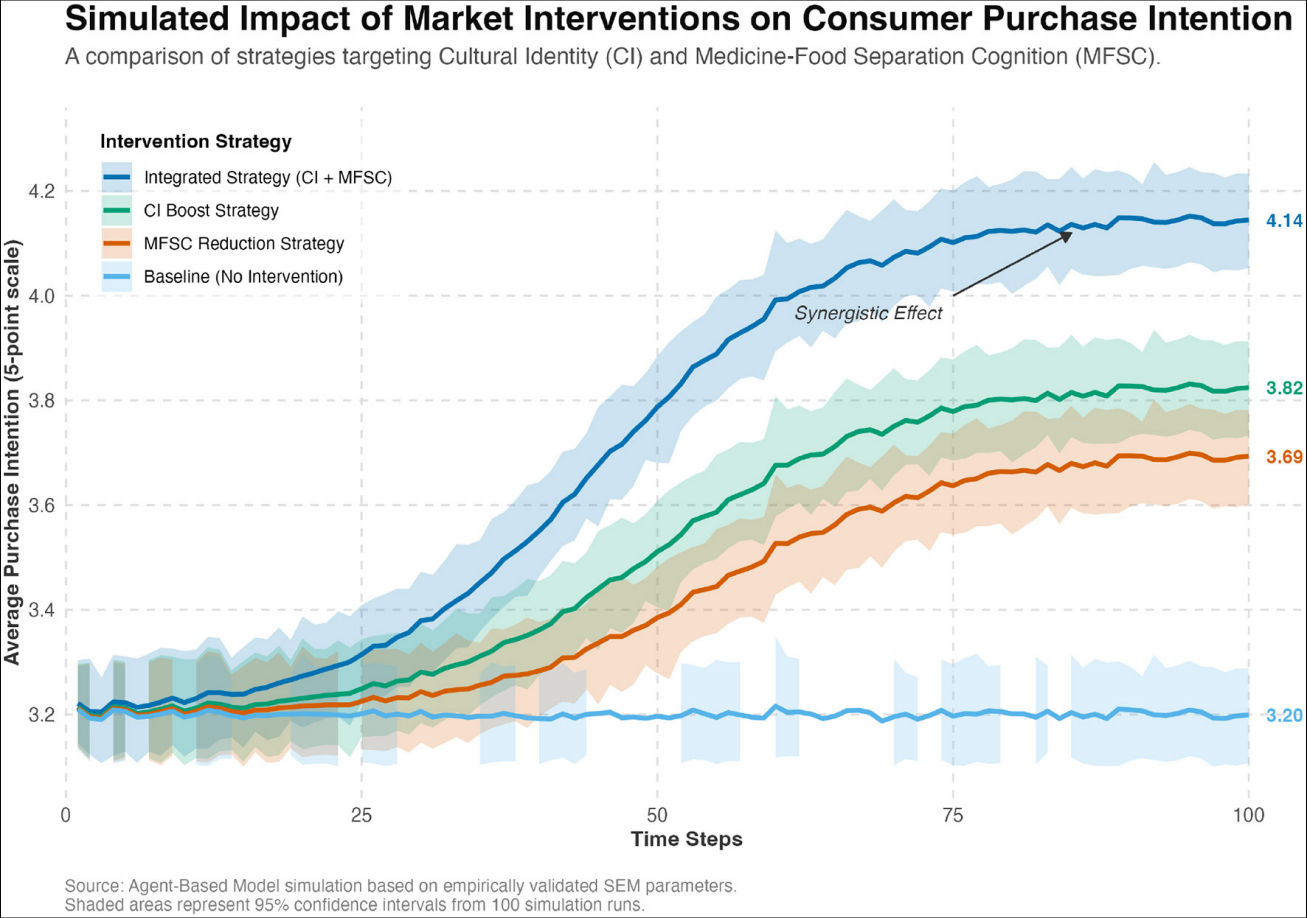
The simulated relationship between increased cultural identity and average repurchase intention (ABM simulation results).

**Figure 9:**
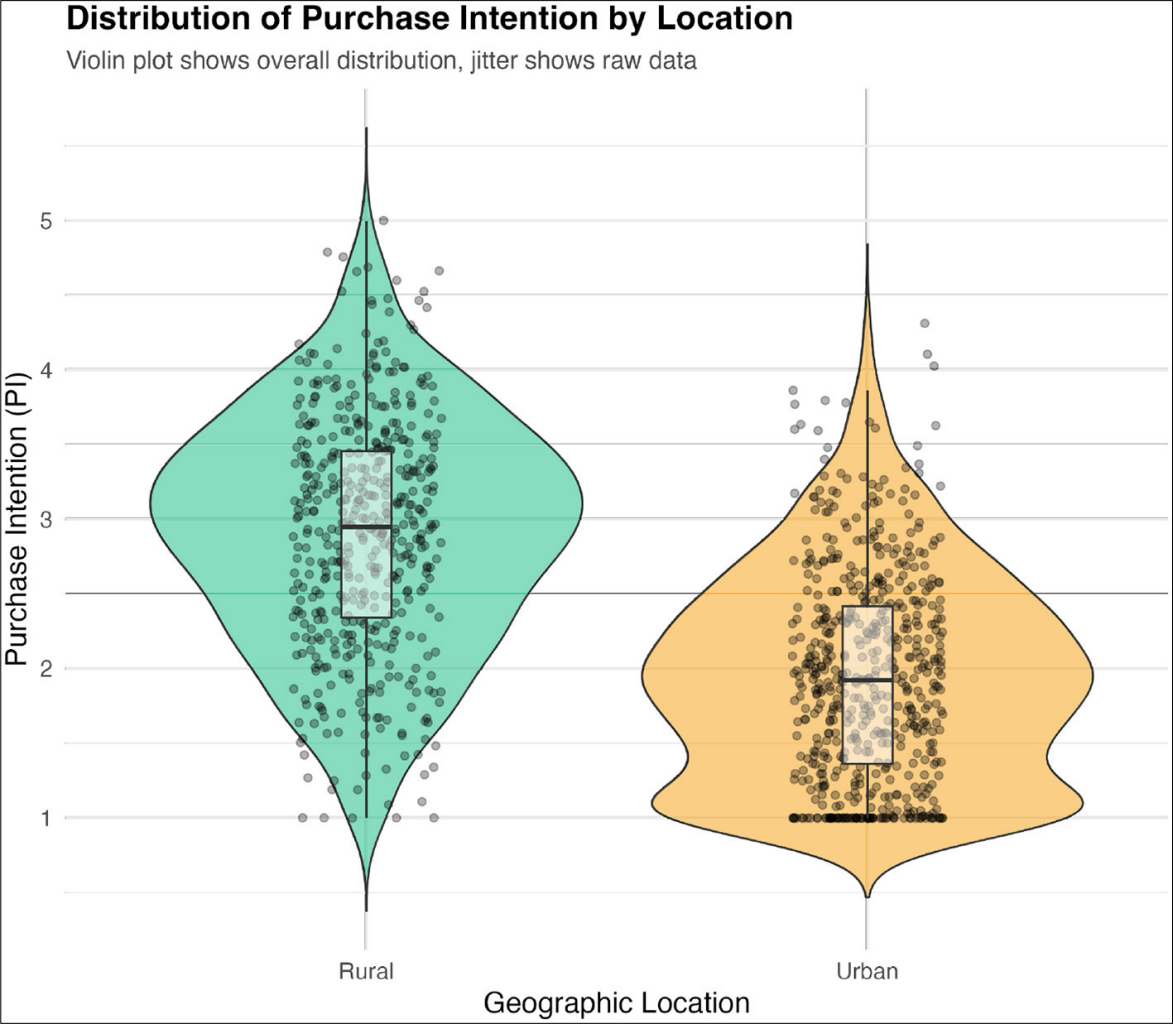
Distribution of purchase intention scores before and after simulated intervention.

ABM simulation was used to explore potential market-level leverage associated with influencing consumer psychology. By operationalizing validated psychometric relationships, simulation (Figure 8) explores scenario where marginal, targeted 0.1-unit enhancement in Cultural Identity of core “26–55” age demographic is associated with 19.7% increase in entire population's mean repurchase intention (from baseline of 3.20–3.83). Violin plot (Figure 9) illustrates that this simulated intervention not only elevates mean intention but also compresses lower tail of distribution, suggesting reduction in number of consumers with very low purchase intent.

## 5 Discussion

### 5.1 Synthesis of findings through triangulation

This study set out to test theoretical model of consumer psychology based on central premise of “Tradition-Modernity Schema.” Integrated CB-SEM-RIA-ABM approach provides strong, triangulated evidence for model's plausibility and utility. CB-SEM confirmed that theoretical structure fits empirical data exceptionally well; RIA quantified relative predictive strength of model's components; and ABM explored their potential dynamic interplay at market level. Convergent findings (Table 9) create coherent narrative, strengthening foundation for theoretical and practical conclusions.

**Table 9:** Triangulation of key findings across methodologies.

**Core insight**	**CB-SEM evidence**	**RIA evidence**	**ABM evidence**
Identification of key conflict	Excellent model fit supports theoretical structure. Identifies significant positive path for CI (β = 0.34) and significant negative path for MFSC (β = −0.29) with medium effect sizes.	Shows that both CI and MFSC are substantial predictors of PI variance.	The model operationalizes this confirmed conflict in agent rules, exploring its potential consequences.
Hierarchy of predictors	Attitude has largest path coefficient (β = 0.44), but direct comparison is limited by multicollinearity.	Quantifies hierarchy: Attitude is strongest predictor (42.8% of *R*^2^), followed by CI (28.1%), MFSC (16.5%), and Price (12.6%).	–
Contextual differences	Multi-group analysis, justified by measurement invariance tests, shows path strengths differ significantly between urban and rural samples with medium-to-large effect size differences.	–	Provides empirical rationale for agent segmentation and exploring targeted interventions.
Potential market leverage	–	–	Explores dynamic scenarios: Simulated intervention (+0.1 CI) is associated with substantial market-level outcome (+19.7% PI), finding robust to sensitivity analysis and network topology.

### 5.2 Theoretical and methodological contributions

Theoretically, this study's primary contribution lies in rigorous conceptualization and initial empirical validation of MFSC construct as measurable psychological barrier to hybrid product acceptance. By situating it within broader “Tradition-Modernity Schema” and systematically differentiating it from related concepts—including trust in science, health literacy, and food neophobia—this research operationalizes specific cognitive factor of “category violation,” highly relevant for understanding consumer resistance to products that blur traditional boundaries (Fiske and Taylor, 2020; Costarelli and Colloca, 2007). Construct therefore offers more precise diagnostic tool than general measures of neophobia, literacy, scientific trust, or health control beliefs, providing both clear theoretical advancement and significant practical utility for marketers and policymakers working with culturally embedded health products.

From a consumer culture theory perspective, the study advances understanding of how cultural identity operates in health product markets. The findings support Social Identity Theory predictions that cultural identity creates direct pathways to purchase intention through identity signaling mechanisms (Scheepers and Ellemers, 2019; Berger and Ward, 2010). This extends existing knowledge by demonstrating that cultural identity effects persist even when controlling for general attitude formation, suggesting that identity-based consumption operates through multiple psychological pathways simultaneously.

Methodologically, “psychometric-to-simulation” pipeline offers replicable template for consumer research that bridges static measurement with dynamic market exploration. Sequence of CB-SEM (to rigorously test theory), RIA (to accurately weigh predictors), and ABM (to explore dynamics) provides more holistic analytical arc than any single method could achieve. This approach moves systematically from identifying what is associated, to quantifying how strongly it is associated, to simulating what might happen if these relationships hold dynamically—progression offering both confirmatory rigor and exploratory insight.

Finally, by interpreting urban-rural divide through lens of Digital Health Ecosystem exposure and Cultural Congruence Theory, this study provides mechanistic explanation for demographic differences going beyond simple geographic categorization. Urban context is modeled not merely as location but as information environment that may systematically strengthen certain cognitive schemas while weakening others (Constantinides, 2022; Lu et al., 2024), providing framework for understanding how modern information ecosystems shape traditional cultural values and their market implications.

### 5.3 Managerial and policy implications

Findings compel shift from monolithic to segmented, data-driven strategies. RIA results clearly establish Attitude as single most important predictor of Purchase Intention (42.8% of explained variance). Therefore, primary strategic objective for marketers should be to foster more positive consumer Attitude. Model suggests two main pathways to achieve this: reinforcing Cultural Identity (CI) and mitigating Medicine-Food Separation Cognition (MFSC).

- Attitude-centric approach for urban markets: in Northern China's urban centers, where MFSC is more significant negative predictor (β=-0.35 vs. β=-0.21 in rural areas), key to building positive Attitude is to lower this cognitive barrier. Marketing must adopt “bridging narratives” that reframe TCM functional foods in modern, scientific, and lifestyle-integrated terms (Salinda et al., 2021). For instance, campaign positioning goji berry extract as “source of zeaxanthin to help filter blue light from screens” does more than just avoid triggering MFSC; by making product's benefits feel relevant and understandable within modern scientific worldview, it directly enhances consumer overall Attitude toward product.
- Attitude-centric approach for rural markets: in rural areas, where Cultural Identity is stronger positive predictor (β = 0.40 vs. β = 0.30 in urban areas), most effective path to positive Attitude is through Cultural Congruence (Salinda et al., 2021; Lu et al., 2024). Marketing emphasizing authenticity, tradition, and local relevance does not just appeal to identity; it leverages that identity to build trust and sense of shared values, which directly translates into more favorable Attitude.
- For policymakers: geographically and generationally contingent nature of health cognition is key insight for “Healthy China 2030” (Tan et al., 2017). Public health education requires tailoring. Urban programs should focus on integrating preventative concepts into scientific framework to bypass MFSC-related biases. Rural programs can focus on validating traditional health knowledge while ensuring product safety and accessibility.

### 5.4 Limitations and future research

This study's conclusions open several avenues for future work. First, its focus on Northern China, while providing regional depth, necessitates future comparative research. Replicating this study in Southern China would provide powerful test of Cultural Congruence theory (Frank et al., 2014).

Second, as explicitly stated, cross-sectional nature of data means analysis identifies plausible associations, not definitive causal chains (Georgeson et al., 2023). Longitudinal studies and experimental designs are necessary next steps to establish causality and determine whether observed associations represent stable individual differences or malleable psychological states.

Third, while comprehensive evidence for MFSC's discriminant validity was provided, continued validation against additional constructs (e.g., Health Consciousness, Traditional Medicine Beliefs) and in different cultural contexts would strengthen its theoretical foundation. Future research should also explore potential mediating variables that might explain the urban-rural differences in MFSC, such as direct measures of digital health platform usage, social media consumption patterns, and exposure to scientific vs. traditional health information sources.

Fourth, self-reported measures may be susceptible to social desirability bias. Future work could complement these with implicit measures like Implicit Association Test (IAT) or behavioral observation methods to capture unconscious attitudes toward TCM functional foods.

Fifth, using urban/rural context as proxy for digital ecosystem immersion represents a simplification. Future research should directly measure digital media consumption patterns, social media health engagement, and control for socioeconomic confounders to better understand mechanisms underlying contextual differences.

Finally, ABM itself can be leveraged as prospective tool for theory development and policy testing. For instance, future models could simulate market competition between firm adopting “heritage” strategy (enhancing CI) and another using “science-bridging” strategy (mitigating MFSC). This would allow in-silico testing of which strategy is more effective under different demographic conditions (Rand and Rust, 2011).

## 6 Conclusion

This research constructed and rigorously tested theoretical model for consumer psychology in TCM functional food market, centered on tension between traditional cultural values and modern cognitive frameworks. Integrated CB-SEM-RIA-ABM methodology empirically supported model's core conflict: Cultural Identity serves as significant positive predictor of purchase intention through both direct identity signaling pathways and indirect attitude formation mechanisms, while newly operationalized “Medicine-Food Separation Cognition” (MFSC) construct represents key negative predictor rooted in categorical thinking patterns. Analysis indicates this tension is context-dependent, with negative association of MFSC being most pronounced among urban consumers exposed to digital health ecosystems. This dynamic represents central strategic consideration for industry.

Methodologically, CB-SEM-RIA-ABM pipeline offers robust template for moving from static psychometric data to dynamic market exploration, providing both confirmatory validation and exploratory insight into potential market interventions. Strategically, findings point toward path for sustainable growth through segmented approaches. By identifying Attitude as primary predictor of purchase intention (42.8% of explained variance), analysis suggests that targeted marketing efforts—specifically those that reinforce cultural identity in rural markets or mitigate cognitive barriers in urban markets to build positive attitude—may be associated with stronger market performance. By untangling complex web of culture, cognition, and context, this study provides evidence-based roadmap for firms navigating this evolving market and for policymakers seeking to promote sustainable consumption that honors heritage while embracing modernity.

## Data Availability

The raw data supporting the conclusions of this article will be made available by the authors, without undue reservation.
